# Identification of Suitable Reference Genes for Gene Expression Studies of Shoulder Instability

**DOI:** 10.1371/journal.pone.0105002

**Published:** 2014-08-14

**Authors:** Mariana Ferreira Leal, Paulo Santoro Belangero, Carina Cohen, Eduardo Antônio Figueiredo, Leonor Casilla Loyola, Alberto Castro Pochini, Marília Cardoso Smith, Carlos Vicente Andreoli, Sintia Iole Belangero, Benno Ejnisman, Moises Cohen

**Affiliations:** 1 Departamento de Ortopedia e Traumatologia, Universidade Federal de São Paulo, São Paulo, São Paulo, Brazil; 2 Disciplina de Genética, Departamento de Morfologia e Genética, Universidade Ferderal de São Paulo, São Paulo, São Paulo, Brazil; 3 Laboratório Interdisciplinar de Neurociência Clínica, Departamento de Psiquiatria, Universidade Federal de São Paulo, São Paulo, São Paulo, Brazil; Florey Institute of Neuroscience & Mental Health, Australia

## Abstract

Shoulder instability is a common shoulder injury, and patients present with plastic deformation of the glenohumeral capsule. Gene expression analysis may be a useful tool for increasing the general understanding of capsule deformation, and reverse-transcription quantitative polymerase chain reaction (RT-qPCR) has become an effective method for such studies. Although RT-qPCR is highly sensitive and specific, it requires the use of suitable reference genes for data normalization to guarantee meaningful and reproducible results. In the present study, we evaluated the suitability of a set of reference genes using samples from the glenohumeral capsules of individuals with and without shoulder instability. We analyzed the expression of six commonly used reference genes (*ACTB*, *B2M*, *GAPDH*, *HPRT1*, *TBP* and *TFRC*) in the antero-inferior, antero-superior and posterior portions of the glenohumeral capsules of cases and controls. The stability of the candidate reference gene expression was determined using four software packages: NormFinder, geNorm, BestKeeper and DataAssist. Overall, *HPRT1* was the best single reference gene, and *HPRT1* and *B2M* composed the best pair of reference genes from different analysis groups, including simultaneous analysis of all tissue samples. GenEx software was used to identify the optimal number of reference genes to be used for normalization and demonstrated that the accumulated standard deviation resulting from the use of 2 reference genes was similar to that resulting from the use of 3 or more reference genes. To identify the optimal combination of reference genes, we evaluated the expression of *COL1A1*. Although the use of different reference gene combinations yielded variable normalized quantities, the relative quantities within sample groups were similar and confirmed that no obvious differences were observed when using 2, 3 or 4 reference genes. Consequently, the use of 2 stable reference genes for normalization, especially *HPRT1* and *B2M*, is a reliable method for evaluating gene expression by RT-qPCR.

## Introduction

Shoulder dislocation occurs in 1 to 2% of the population [1], and traumatic injuries account for 95% of shoulder dislocation episodes [2]. These shoulder injuries are frequently observed in young athletes that are involved in competitive sports [3], and shoulder instability (SI) is often observed after the initial episode of shoulder dislocation, with a recurrence rate of up to 100% in young athletes [4], [5].

After episodes of shoulder dislocation, SI patients present plastic deformation of the glenohumeral capsule [6], [7]. Although the antero-inferior (AI) region of the capsule is the most frequently injured site [7], [8], previous macroscopic analysis of the collagen fiber bundle architecture in the AI region of the glenohumeral capsule revealed that a system of bundles spirally crossing one another permits the entire capsule to resist tensile and shear loads [9]. As a result, there is a reciprocal load-sharing relationship within the capsule whereby tensile load in either the anterior or superior structures is concomitant with laxity in the posterior (P) or inferior portion, respectively [7], suggesting that different portions of the capsule may be modified in traumatic anterior SI cases.

Currently, little is known about capsule biology, especially in patients with SI. An improved understanding of the underlying biology will be important for guiding patient management and development of new therapeutic options that will be complementary to surgery. Our group recently began investigating alterations in gene expression in SI, as gene expression analysis has previously been used to increase understanding of the molecular events involved in other traumatic sport injuries such as ligament [10], [11] and tendon injuries (for a review, see [12]).

As a result of its accuracy, sensitivity and capacity for high throughput analysis, reverse-transcription quantitative polymerase chain reaction (RT-qPCR) is currently considered to be the gold standard technique for evaluation of gene expression [13]; furthermore, this technique is commonly used to validate data obtained by other methods [14].

To obtain reliable data using RT-qPCR, gene expression levels must be normalized using internal controls within each sample [15]. The use of one or more reference genes can correct biases caused by variations in the complementary DNA (cDNA) input or the efficiency of reverse transcription or amplification. Ideally, reference genes should be stably expressed or at least vary only slightly in expression in all tissues or cells under the conditions of the experiment [16].

Although several genes are commonly used as controls (e.g., *GAPDH* and *ACTB*), they can also be regulated and expressed at varying levels [17]. Because the quality of data from gene expression analyses is affected by the quality of reference genes used, it is recommended that reference gene expression stability be validated for each target tissue and disease [18], [19].

The suitability of reference genes has been evaluated in some human musculoskeletal disease such as osteoarthritic articular cartilage (hip and knee) [20], human lumbar vertebral endplate with modic changes [21] and skeletal muscle with chronic degenerative changes [22]. However, to our knowledge no previous studies have identified the best individual or set of reference genes for gene expression analysis from samples of shoulder capsules.

In this study, we assessed the suitability of six reference genes frequently used in the literature (*ACTB*, *B2M*, *GAPDH*, *HPRT1*, *TBP* and *TFRC*) using samples from 3 sites within the glenohumeral capsule [AI, antero-superior (AS) and P portions] of SI patients and control individuals by analyzing gene stability using 4 freely available software packages.

## Materials and Methods

### Patients

We tested tissue samples from 13 patients with traumatic anterior SI from São Paulo Hospital of the Federal University of São Paulo (UNIFESP), Brazil. All of the patients were treated with shoulder immobilization for a minimum of 2 weeks following the first episode of shoulder dislocation and underwent arthroscopic surgical treatment for SI.

Additionally, 5 patients who underwent arthroscopically assisted treatment for acromioclavicular dislocation were included in this study as a control group. These patients did not present with any history of SI or signs of SI injury under anesthesia; furthermore, we did not find any radiological indications of glenohumeral capsule alterations. All control patients were physically active. Table 1 displays the main clinical outcomes of the studied cases and controls.

**Table 1:** Distribution of the clinical outcomes of shoulder instability patients and controls.

Variable	Cases	Controls
Age at surgery, years (mean ± SD)	28.42±6.21	30.35±14.36
Gender (% of male)	92.3%	100%
Age of onset, years (mean ± SD)	25.83±7.38	
Duration of condition, years (mean ± SD)	2.7±3.03	
Duration of condition (% of >1 year)	84.6%	
Number of injuries (% of >1 dislocation episode)	69.2%	

The study was approved by the ethics committee of the UNIFESP. Written informed consent with approval of the ethics committee was obtained from all patients prior to specimen collection.

### Tissue samples

During the arthroscopic procedures, tissue samples were obtained from the AI, AS and P sites of the glenohumeral capsule of each patient. Biopsy samples from the AI and AS sites were obtained using the scope in the posterior portal and the basket grasper in the anterior portal. The AI specimen was taken from the most inferior region of the glenohumeral capsule next to the inferior glenohumeral ligament, while the AS specimen was taken in the direction of the anterior portal below the biceps tendon, in the rotator interval area. The P specimen was taken in the direction of the posterior portal during evaluation of the posterior capsulolabral complex with the scope in the anterior portal and the basket grasper in the posterior portal.

All tissue specimens were immediately immersed in RNAlater solution (Qiagen, Germany) and stored at -20°C until RNA extraction.

### RNA extraction and cDNA synthesis

Total RNA was extracted using an RNeasy mini kit (Qiagen, Germany) according to the manufacturer's protocol. RNA concentration and quality were determined using a Nanodrop ND-1000 (Thermo Scientifc, USA) and the integrity of the RNA was verified by gel electrophoresis on a 1% agarose gel. cDNA was synthesized from 60–100 ng of RNA using a High-Capacity cDNA Archive kit (Life Technologies, USA) according to the manufacturer's protocol.

### RT-qPCR

To detect the range of expression of the six candidate reference genes, reactions were performed in triplicate using TaqMan inventoried Assays-on-Demand probes (Life Technologies, USA) and the Applied Biosystems 7500 fast real-time PCR system.

To identify the best combination of reference genes, we also quantified the mRNA expression of a target gene, *COL1A1* using the candidate reference genes for normalization. *COL1A1* was select as a target gene since it codified the α1 chain of human procollagen type I, which is the most prominent protein of the capsule [23]. In addition, upregulation of *COL1A1*, as well as other collagen genes and their protein products, has been reported in several joint injuries, including injured Achilles tendon[12], [24], anterior cruciate ligament[25], [26], [27] and rotator cuff tear[11], [28].

For each sample, candidate reference and target genes were run on the same plate to exclude technical variations. The 6 reference genes and target gene are summarized in Table 2.

**Table 2:** Summary of six reference genes and a target gene.

Gene symbol	Name	Accesion number	Assay*	Gene function
*ACTB*	Beta-actin	NM_001101.2	4352935E	Cytoskeletal structural protein
*B2M*	Beta-2-microglobulin	NM_004048.2	4333766T	Beta-chain of major histocompatibility complex class I molecules
*GAPDH*	Glyceraldehyde-3-phosphate dehydrogenase	NM_002046.3	4352934E	Oxidoreductase in glycolysis and gluconeogenesis
*HPRT1*	Hypoxanthine phosphoribosyl-transferase	NM_000194.1	4333768T	Purine synthesis in salvage pathway
*TBP*	TATA box binding protein	NM_003194.3	4333769T	RNA polymerase II, transcription factor
*TFRC*	Transferrin receptor (CD71)	NM_003234.1	4333770T	Cellular iron ion homeostasis
*COL1A1*	Collagen, type I, alpha 1	NM_000088.3	Hs00164004_m1	Extracellular matrix structural protein

*TaqMan probes were purchased as assays-on-demand products for gene expression (Life Technologies, USA).

The expression of *COL1A1* across the samples was calculated using the equation 2^(−ΔCt)^, in which [ΔCt (cycle threshold)  =  target gene (collagen) Ct – geometric mean of reference genes Ct].

### Analysis of reference gene expression stability

We categorized the tissue samples into the following 12 groups: 1) AI samples from cases (SI patients); 2) AS samples from cases; 3) P samples from cases; 4) all tissue samples from cases; 5) AI samples from controls; 6) AS samples from controls; 7) P samples from controls; 8) all tissue samples from controls; 9) all AI samples; 10) all AS samples; 11) all P samples; and 12) all tissue samples.

For comparisons of candidate reference gene stability we used NormFinder (http://www.mdl.dk/publicationsnormfinder.htm), geNorm (http://medgen.ugent.be/~jvdesomp/genorm/http://medgen.ugent.be/~jvdesomp/genorm/), BestKeeper1 (http://www.gene-quantifcation.de/bestkeeper.html) and DataAssist (http://www.lifetechnologies.com/us/en/home/technical-resources/software-downloads/dataassist-software.html) software programs according to the recommendations of the software guides. NormFinder accounts for both intra- and inter-group variations when evaluating the stability of each single reference gene and assigns lower stability values to the genes that are most stably expressed [29]. geNorm calculates the expression stability value (M) for each gene based on the average pairwise expression ratio between a particular gene and all other reference genes. The most stably expressed gene yields the lowest M value, and then the two most stable reference genes are determined by stepwise exclusion of the least stable gene [15]. Bestkeeper was used to rank the 6 reference genes based on the standard deviation (SD) and coefficient of variance (CV) expressed as a percentage of the cycle threshold (Ct) level [30]. Lastly, DataAssist software provided a metric to measure reference gene stability based on the geNorm algorithm. In contrast to the other programs, DataAssist uses RQ to calculate the stability value of individual candidate reference genes.

GenEx software (http://genex.gene-quantifcation.info/) was used to determine the optimal number of reference genes by calculating the accumulated standard deviation (Acc.SD).

## Results

### Reference gene expression levels

**Figure 1:**
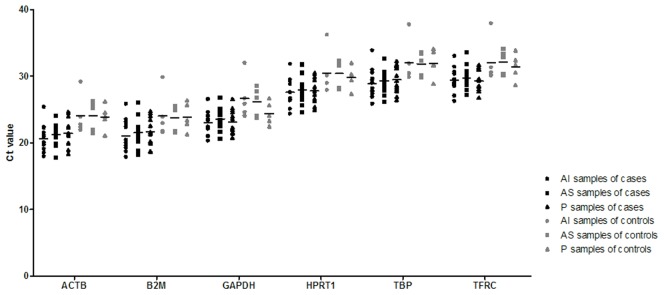
RT-qPCR detection of the expression levels of six reference genes. A lower cycle threshold value (Ct) indicates higher gene expression. AI: antero-inferior region of the glenohumeral capsule; AS: antero-superior region of the glenohumeral capsule; P: posterior region of the glenohumeral capsule.

The distribution of Ct values for each of the 6 candidate reference genes is shown in Figure 1. These genes displayed a wide range of expression levels. *ACTB* (mean Ct value ± SD  = 21.91±2.327) followed by *B2M* (22.08±2.436) presented the highest expression levels. In contrast, *TFRC* (30.11±2.125) and *TBP* (29.95±2.358) presented the lowest expression levels in glenohumeral capsule samples.

### Reference gene expression stability

Table 3 displays the stability value ranking of the single candidate reference genes as determined by the different software packages. In our analysis, all reference genes for all analysis groups presented M values less than the geNorm threshold of 1.5 that is recognized as stable.

**Table 3:** Ranking of the candidate single reference genes by each software package used.

NormFinder		geNorm		BestKeeper		DataAssist	
Stability value*	Ranking	M value*	Ranking	CV*	Ranking	Score*	Ranking
AI samples of cases							
0.193	*HPRT1*	0.419	*TFRC*	4.59	*TFRC*	0.782	*GAPDH*
0.350	*ACTB*	0.419	*GAPDH*	5.55	*TBP*	0.814	*HPRT1*
0.414	*TFRC*	0.488	*HPRT1*	5.57	*GAPDH*	0.8355	*ACTB*
0.435	*GAPDH*	0.503	*ACTB*	6.15	*HPRT1*	1.055	*TBP*
0.599	*B2M*	0.572	*B2M*	7.16	*ACTB*	1.131	*TFRC*
0.607	*TBP*	0.624	*TBP*	9.13	*B2M*	1.467	*B2M*
AS samples of cases							
0.276	*HPRT1*	0.246	*HPRT1*	4.10	*TFRC*	0.768	*GAPDH*
0.293	*GAPDH*	0.246	*B2M*	4.43	*TBP*	0.823	*HPRT1*
0.463	*TBP*	0.444	*GAPDH*	4.72	*HPRT1*	0.869	*ACTB*
0.478	*B2M*	0.528	*TBP*	5.06	*GAPDH*	0.923	*TFRC*
0.486	*TFRC*	0.562	*TFRC*	5.18	*ACTB*	0.933	*TBP*
0.593	*ACTB*	0.611	*ACTB*	6.39	*B2M*	1.611	*B2M*
P samples of cases							
0.179	*HPRT1*	0.337	*B2M*	4.41	*TFRC*	0.861	*HPRT1*
0.468	*B2M*	0.337	*ACTB*	5.39	*HPRT1*	0.987	*TBP*
0.520	*ACTB*	0.359	*HPRT1*	5.91	*TBP*	1.087	*GAPDH*
0.521	*TBP*	0.488	*TBP*	6.76	*GAPDH*	1.101	*ACTB*
0.610	*TFRC*	0.619	*TFRC*	7.70	*ACTB*	1.193	*TFRC*
0.933	*GAPDH*	0.752	*GAPDH*	7.88	*B2M*	1.462	*B2M*
All samples of cases							
0.134	*HPRT1*	0.377	*HPRT1*	4.36	*TFRC*	0.836	*HPRT1*
0.224	*B2M*	0.377	*B2M*	5.36	*TBP*	0.913	*GAPDH*
0.246	*TBP*	0.523	*ACTB*	5.50	*HPRT1*	0.958	*ACTB*
0.254	*ACTB*	0.599	*TBP*	5.87	*GAPDH*	1.012	*TBP*
0.251	*GAPDH*	0.666	*TFRC*	6.83	*ACTB*	1.091	*TFRC*
0.275	*TFRC*	0.705	*GAPDH*	7.92	*B2M*	1.493	*B2M*
AI samples of controls							
0.021	*TBP*	0.215	*TBP*	7.10	*TBP*	0.787	*HPRT1*
0.204	*HPRT1*	0.215	*HPRT1*	7.44	*TFRC*	0.798	*ACTB*
0.279	*ACTB*	0.321	*ACTB*	7.61	*HPRT1*	0.943	*TBP*
0.353	*B2M*	0.389	*B2M*	8.15	*GAPDH*	0.953	*GAPDH*
0.447	*GAPDH*	0.410	*GAPDH*	8.58	*ACTB*	1.110	*TFRC*
0.556	*TFRC*	0.471	*TFRC*	9.70	*B2M*	2.036	*B2M*
AS samples of controls							
0.265	*B2M*	0.221	*HPRT1*	4.85	*TFRC*	1.063	*HPRT1*
0.358	*HPRT1*	0.221	*B2M*	4.79	*TBP*	1.144	*GAPDH*
0.402	*TBP*	0.364	*TBP*	6.13	*HPRT1*	1.180	*ACTB*
0.436	*ACTB*	0.470	*GAPDH*	6.92	*GAPDH*	1.199	*TFRC*
0.458	*GAPDH*	0.530	*ACTB*	7.23	*B2M*	1.249	*TBP*
0.491	*TFRC*	0.561	*TFRC*	7.94	*ACTB*	1.4613	*B2M*
P samples of controls							
0.263	*TBP*	0.617	*TFRC*	3.85	*HPRT1*	1.301	*TBP*
0.303	*TFRC*	0.617	*TBP*	4.69	*TFRC*	*1.351*	*HPRT1*
0.574	*HPRT1*	0.685	*HPRT1*	4.73	*TBP*	1.582	*ACTB*
0.876	*B2M*	0.801	*B2M*	5.20	*ACTB*	1.639	*GAPDH*
0.946	*ACTB*	0.893	*ACTB*	5.66	*GAPDH*	1.702	*TFRC*
1.335	*GAPDH*	1.064	*GAPDH*	7.06	*B2M*	1.887	*B2M*
All samples of controls							
0.264	*HPRT1*	0.460	*TBP*	5.44	*TBP*	1.164	*TBP*
0.345	*TFRC*	0.460	*B2M*	5.62	*TFRC*	1.190	*HPRT1*
0.370	*TBP*	0.573	*ACTB*	5.77	*HPRT1*	1.244	*ACTB*
0.422	*ACTB*	0.637	*HPRT1*	7.25	*ACTB*	1.457	*GAPDH*
0.457	*B2M*	0.667	*TFRC*	7.27	*GAPDH*	1.599	*TFRC*
0.731	*GAPDH*	0.843	*GAPDH*	7.86	*B2M*	1.812	*B2M*
All AI samples							
0.169	*TBP*	0.467	*GAPDH*	5.33	*TFRC*	0.833	*HPRT1*
0.207	*HPRT1*	0.467	*ACTB*	6.70	*TBP*	0.880	*GAPDH*
0.244	*ACTB*	0.541	*HPRT1*	6.99	*HPRT1*	0.852	*ACTB*
0.250	*B2M*	0.577	*TBP*	7.64	*GAPDH*	1.044	*TBP*
0.388	*GAPDH*	0.608	*B2M*	8.91	*ACTB*	1.161	*TFRC*
0.400	*TFRC*	0.631	*TFRC*	9.90	*B2M*	1.658	*B2M*
All AS samples							
0.118	*HPRT1*	0.270	*HPRT1*	5.02	*TBP*	0.891	*HPRT1*
0.125	*B2M*	0.270	*B2M*	5.19	*TFRC*	0.932	*GAPDH*
0.143	*GAPDH*	0.460	*GAPDH*	5.93	*HPRT1*	0.998	*ACTB*
0.154	*TBP*	0.517	*TBP*	6.24	*GAPDH*	1.045	*TBP*
0.177	*TFRC*	0.555	*TFRC*	6.93	*ACTB*	1.046	*TFRC*
0.180	*ACTB*	0.598	*ACTB*	7.62	*B2M*	1.549	*B2M*
All P samples							
0.183	*TFRC*	0.489	*B2M*	5.45	*TFRC*	1.000	*HPRT1*
0.191	*HPRT1*	0.489	*ACTB*	5.94	*HPRT1*	1.075	*TBP*
0.222	*TBP*	0.611	*HPRT1*	6.48	*TBP*	1.244	*ACTB*
0.294	*B2M*	0.633	*TBP*	6.59	*GAPDH*	1.27	*GAPDH*
0.328	*ACTB*	0.710	*TFRC*	8.54	*B2M*	1.322	*TFRC*
0.471	*GAPDH*	0.864	*GAPDH*	8.61	*ACTB*	1.546	*B2M*
All samples							
0.056	*HPRT1*	0.494	*HPRT1*	5.31	*TFRC*	0.926	*HPRT1*
0.094	*TBP*	0.494	*B2M*	6.12	*TBP*	1.079	*ACTB*
0.098	*TFRC*	0.594	*TBP*	6.25	*HPRT1*	1.087	*TBP*
0.119	*ACTB*	0.626	*ACTB*	6.88	*GAPDH*	1.114	*GAPDH*
0.119	*B2M*	0.678	*TFRC*	8.13	*ACTB*	1.1927	*TFRC*
0.199	*GAPDH*	0.756	*GAPDH*	8.68	*B2M*	1.586	*B2M*

*A lower value indicates higher stability in gene expression. AI: antero-inferior region of the glenohumeral capsule; AS: antero-superior region of the glenohumeral capsule; P: posterior region of the glenohumeral capsule.

For most of the analysis groups, the various software packages suggested different single best reference genes, and all four software packages generated different rankings of reference gene stability for each analysis group.

Typically, gene expression studies compare transcript levels between case (i.e., the injured tissue) and control samples. When considering the AI samples, no single gene was repeatedly identified as being the best reference gene by the various software packages. In contrast, NormFinder, geNorm and DataAssist each identified *HPRT1* as the most stable gene in AS samples, and NormFinder and BestKeeper both identified *TFRC* as the most stable gene in P samples.

In some studies, researchers have investigated a possible association between gene expression and clinical variables. In the present study, *HPRT1*, followed by *B2M* was the most suitable reference gene for the different tissue categories from cases. For the tissue categories from controls, *TBP* and *HPRT1* were the most stable reference genes.

When all 54 samples were considered, *HPRT1* and *B2M* (M value  = 0.494) were identified as the most stably expressed reference genes by geNorm, and *HPRT1* was also identified as the most stable reference gene by the NormFinder and DataAssist software. Moreover, *HPRT1* was the gene most frequently identified as a suitable reference gene when considering all the analysis groups.

Table 4 displays the best combinations of reference genes as suggested by the 4 software packages. Overall, *HPRT1* and *B2M* were the most suitable reference genes, and this pair of genes was the most frequently identified when evaluating all cases or all samples, as well as when evaluating only AS samples. In contrast, *GAPDH* and *ACTB* was the most frequently identified pair from the analysis of AI samples; *ACTB* and *B2M* was the most frequently identified pair from the analysis of P samples; and *TBP* and *B2M* was the most frequently identified pair of reference genes when all controls were evaluated simultaneously.

**Table 4:** Best pair of reference genes according to each software for each group of sample.

Samples	Best pair of reference genes by software			
	NormFinder	geNorm	BestKeeper	DataAssist
AI samples of cases	*HPRT1* + *ACTB*	*TFRC* + *GAPDH*	*HPRT1* + *B2M*	*GAPDH* + *ACTB*
AS samples of cases	*HPRT1* + *GAPDH*	*HPRT1* + *B2M*	*HPRT1* + *B2M*	*HPRT1* + *GAPDH*
P samples of cases	*HPRT1* + *B2M*	*ACTB* + *B2M*	*HPRT1* + *B2M*	*HPRT1* + *ACTB*
All samples of cases	*HPRT1* + *B2M*	*HPRT1* + *B2M*	*HPRT1* + *B2M*	*HPRT1* + *ACTB*
AI samples of controls	*HPRT1* + *TBP*	*HPRT1* + *TBP*	*TBP* + *ACTB*	*HPRT1* + *ACTB*
AS samples of controls	*HPRT1* + *B2M*	*HPRT1* + *B2M*	*HPRT1* + *B2M*	*TBP* + *TFRC*
P samples of controls	*TBP* + *TFRC*	*TBP* + *TFRC*	*TBP* + *TFRC*/*TBP* + *B2M*	*TBP* + *TFRC*
All samples of controls	*HPRT1* + *TFRC*	*TBP* + *B2M*	*TBP* + *B2M*	*HPRT1* + *TBP*
All AI samples	*HPRT1* + *TBP*	*GAPDH* + *ACTB*	*HPRT1* + *B2M*	*GAPDH* + *ACTB*
All AS samples	*HPRT1* + *B2M*	*HPRT1* + *B2M*	*HPRT1* + *B2M*	*GAPDH* + *ACTB*
All P samples	*HPRT1* + *TFRC*	*ACTB* + *B2M*	*ACTB* + *B2M*	*TBP* + *TFRC*
All samples	*HPRT1* + *TBP*	*HPRT1* + *B2M*	*HPRT1* + *B2M*	*HPRT1* + *ACTB*

AI: antero-inferior region of the glenohumeral capsule; AS: antero-superior region of the glenohumeral capsule; P: posterior region of the glenohumeral capsule.

**Figure 2:**
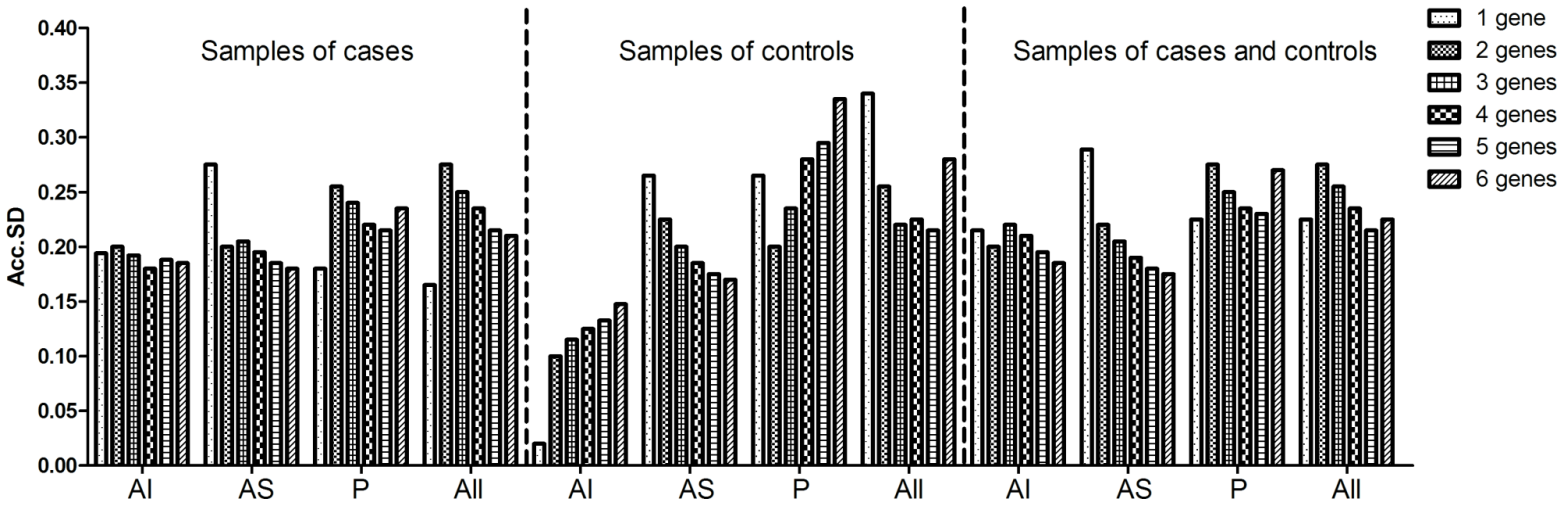
Accumulated standard deviation for the 6 reference genes in glenohumeral capsule samples. Lower values of accumulated standard deviation (Acc.SD) indicate the optimal number of reference gene as estimated by the GenEx software package. AI: antero-inferior region of the glenohumeral capsule; AS: antero-superior region of the glenohumeral capsule; P: posterior region of the glenohumeral capsule.

The 4 software packages only indicated up to 2 genes as the best combination of reference genes. We used the GenEx software package to determine if reliable normalization would require more than 2 reference genes. In this analysis the optimal number of reference genes is indicated by the lowest SD, and with the exception of the analysis of P site samples from controls, the Acc.SD of 2 reference genes did not differ more than 0.1 from the observed metric when using more than 2 genes (Figure 2).

### Effects of reference gene choice

To validate the selection of the appropriate reference genes for normalization, an expression analysis was performed comparing data from samples of patients with shoulder instability to controls for the three capsule sites. This analysis was performed using *COL1A1* as a target gene in all the analyses. As reference genes, we used the most frequently identified pairs described above. We also performed the *COL1A1* expression analysis using 3 reference genes (*HPRT1* + *B2M* + *ACTB*) and 4 reference genes (*HPRT1* + *B2M* + *ACTB* + *TBP*).

**Figure 3:**
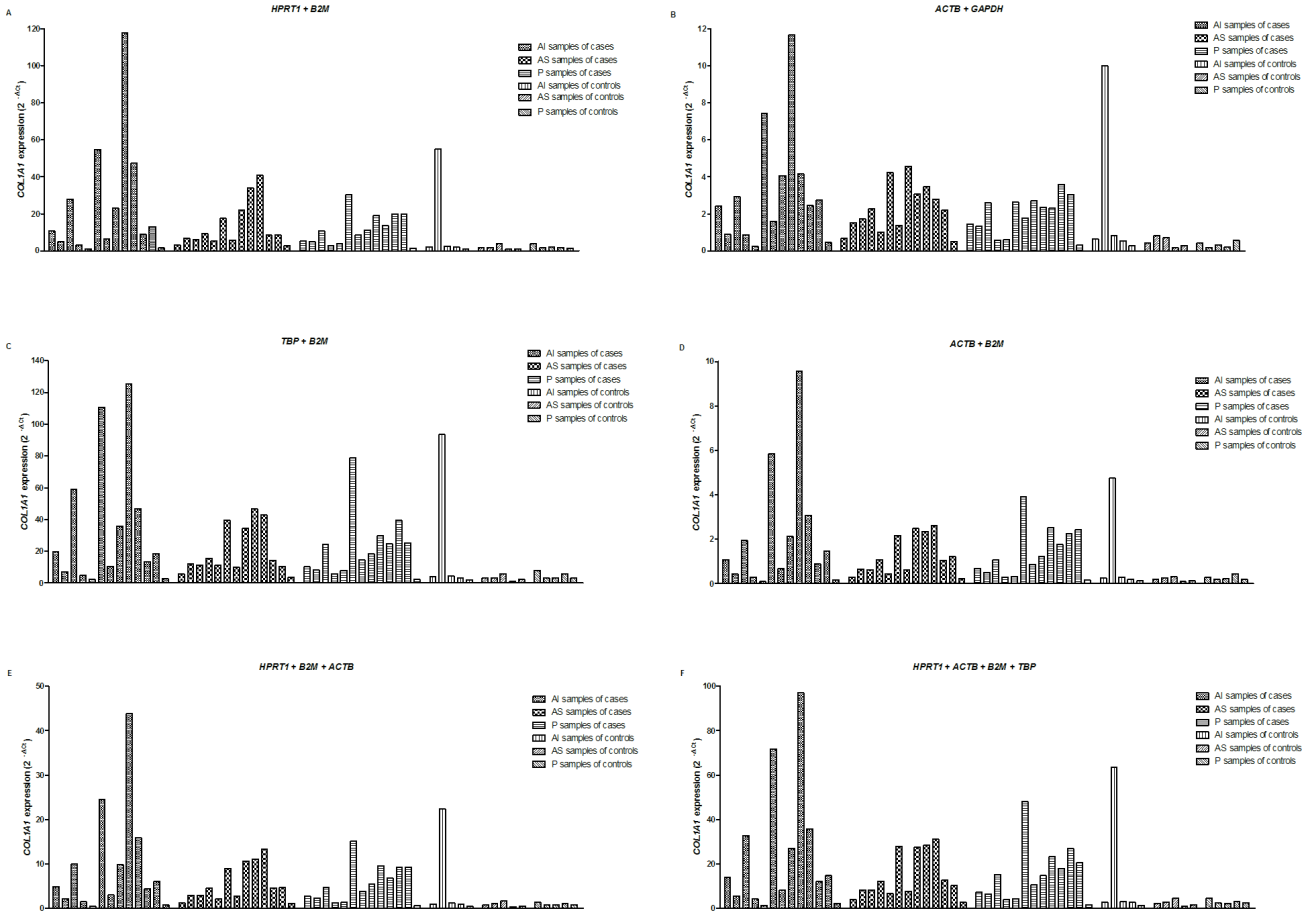
*COL1A1* expression normalized by different combinations of candidate reference genes in glenohumeral capsule specimens. AI: antero-inferior region of the glenohumeral capsule; AS: antero-superior region of the glenohumeral capsule; P: posterior region of the glenohumeral capsule.

Although the normalized expression quantities differed between the various combinations of reference genes, the distributions of *COL1A1* expression in the studied samples were similar (Figure 3). Moreover, *COL1A1* expression was significantly increased in the AS and P sites of the glenohumeral capsule of cases compared to the controls using all the reference genes combinations described above (p<0.05 for all analyses using the Mann-Whitney test; Table 5). Regardless of the reference gene combination used, *COL1A1* expression in the capsule AI site did not differ between cases and controls (p>0.05 using the Mann-Whitney test for all analyses; Table 5).

**Table 5:** *COL1A1* expression normalized by different combinations of reference genes in the glenohumeral capsule of patients with shoulder instability and controls.

Reference genes	*COL1A1* expression								
	AI			AS			P		
	Cases (median ± IQR)	Controls (median ± IQR)	p-value	Cases (median ± IQR)	Controls (median ± IQR)	p-value	Cases (median ± IQR)	Controls (median ± IQR)	p-value
*HPRT1* + *B2M*	10.79±33.54	2.13±27.24	0.173	8.47±14.31	1.81±1.92	0.001*	10.77±15.08	1.86±1.30	0.007*
*ACTB* + *GAPDH*	2.45±3.23	0.65±4.99	0.208	2.20±2.07	0.42±0.53	0.003*	2.33±1.69	0.33±0.29	0.002*
*TBP* + *B2M*	18.39±46.76	3.95±46.42	0.143	11.99±29.76	3.10±3.02	0.001*	18.32±19.27	3.24±3.86	0.014*
*ACTB* + *B2M*	1.09±2.23	0.25±2.36	0.153	1.06±1.71	0.19±0.18	0.002*	1.08±1.91	0.22±0.17	0.014*
*HPRT1* + *B2M* + *ACTB*	4.91±11.07	1.04±11.07	0.173	4.57±7.42	0.82±0.86	0.001*	4.81±7.43	0.91±0.44	0.007*
*HPRT1* + *ACTB* + *B2M* + *TBP*	14.12±29.27	2.9±31.30	0.143	10.29±20.54	2.35±2.35	0.001*	14.71±16.46	2.48±1.47	0.010*

AI: antero-inferior region of the glenohumeral capsule; AS: antero-superior region of the glenohumeral capsule; P: posterior region of the glenohumeral capsule; IQR: interquartile range. *p<0.05 by Man-Whitney test.

## Discussion

Our group recently began investigating the molecular alterations involved in shoulder instability and other orthopedic lesions. We hypothesized that misregulated expression of several genes may have a role in the capsular deformation observed in SI patients and that such molecular alterations may explain the high rate of shoulder dislocation recurrence after the first episode of traumatic dislocation. Additionally, an increased understanding of gene expression modification in response to injury may aid in determination of patient prognosis and in the development of new treatment strategies.

RT-qPCR is one of the most commonly utilized approaches in functional genomics research, and its use in gene expression analysis may become routine. However, many authors do not critically evaluate their RT-qPCR experiments, and as a result, the experiments are improperly designed and difficult to repeat due to insufficient data quality [31]. To minimize the influence of differences between samples in the extraction of mRNA, reverse transcription and PCR [17], is necessary to normalize target gene expression by a known factor. Consequently, the use of suitable reference genes with stable expression in the studied tissue (normal and/or injured) is essential for effective data normalization and the acquisition of accurate and meaningful biological data.

Reference genes have been described for RT-qPCR studies in several diseases and tissues [20], [21], [22], [32], [33], [34], [35], and our group recently identified the most stable reference genes in gastric neoplastic and non-neoplastic samples, as well as in gastric cancer cell lines [36]. To the best of our knowledge, no prior study has sought to identify suitable reference genes for gene expression analysis in the glenohumeral capsule.

In the present study, we used 4 software packages (NormFinder, geNorm, BestKeeper, and DataAssist) to evaluate the stability of reference gene expression. Each software package uses distinct algorithms, and as a result, different results can be expected. Therefore, it is important to use more than one software package to identify the most suitable reference genes among a set of candidates. Although the 4 software packages differed in their rankings of reference gene stability as well as in the identity of the most suitable pair, at least two programs produced results that agreed for almost all the analyses. Our results demonstrate that the use of 4 statistical tools aids the identification of the best reference genes.

In the different groups of analyses, *HPRT1* seems to be the most suitable gene overall; however, it is increasingly clear that in most situations a single reference gene is not sufficiently stable [37]. When a larger number of reference genes is used, the SD of the normalization factor (mean of reference gene expression) is reduced and the random variation among the expression of tested is partially cancelled.

Using the GenEx software, we observed that the Acc.SD of 2 reference genes differed no more than 0.1 from that observed in most of the analysis groups when 3, 4, 5 or 6 reference genes were used. Inclusion of additional reference genes increases the time and money required for analysis; therefore, it is important to consider the degree of improvement and overall noise contributed by reference genes when deciding how many reference genes are required. Considering that the reproducibility of real-time PCR equipment is rarely less than 0.1 cycle (estimated as SD of technical replicates), we believe that the use of more than two reference genes does not significantly improve the data quality.

Only for the analysis of the control glenohumeral capsule P site was the Acc.SD using 6 genes higher than 0.1 in comparison to the use of 2 reference genes, meaning that the use of fewer reference genes is also the most appropriate for this sample group.

Although different pairs of reference genes were determined to be the most suitable for the various analysis groups, the combination of *HPRT1* and *B2M* was the most frequently identified pair. Furthermore, our results demonstrated that *HPRT1* and *B2M* was the best gene pair for comparisons requiring the use of a combination of reference genes for analysis of samples of different portions of the glenohumeral capsule from patients with and without SI.

To identify the best combination of reference genes, we evaluated *COL1A1* expression in samples from the AI, AS and P sites of the glenohumeral capsules of cases and controls. To normalize *COL1A1* expression, we paired *HPRT1* and *B2M*; *GAPDH* and *ACTB*; *ACTB* and *B2M* and *TBP* and *B2M*. Furthermore we normalized *COL1A1* expression using the 3 and 4 most stable genes to evaluate the effects of increasing the number of reference genes. Within the case and control groups, no obvious differences in *COL1A1* expression were observed when normalized with different combinations of reference genes. Moreover, statistical comparison revealed that *COL1A1* expression differed between the case and control samples from the AS and P capsule portions independently of which reference gene combination was used for normalization.

Therefore, our results show that combinations of 2 genes can be used for the analysis of glenohumeral capsule samples and that it is not necessary to use 3 or more reference genes. However, it should be noted that all the reference genes presented an M value less than the geNorm threshold of 1.5 recognized as stable under the different experimental conditions tested.

Our study presented some limitations. First, we only included a limited number of candidate reference genes, and it is likely that some other genes may be also used as internal references for gene expression studies in glenohumeral capsule samples from patients with or without history of shoulder dislocation. Second, our results only apply directly to glenohumeral capsule. It is unclear how well our results could be extended to other joint capsules. Therefore, when new cohorts of tissue samples are used, we suggest performing specific gene expression studies, in order to identify the most stable reference genes to be used for normalization. However, it is important to highlight that our results may be relevant to the study of SI, as well as to the study of the normal glenohumeral capsule.

## Conclusions

In the present study, we evaluated the suitability of reference genes using samples of glenohumeral capsules from individuals with and without history of shoulder dislocation episodes. Examining the different analysis groups, *HPRT1* appears to be the most suitable reference gene. We observed that 2 reference genes, especially *HPRT1* and *B2M*, might be used in combination for accurate normalization of RT-qPCR data in studies of molecular alterations in the glenohumeral capsule of SI patients. The results of this work may benefit future studies of the glenohumeral capsule that require more accurate gene expression quantification in this tissue.
